# Protocol for viral vector-mediated measurement of transcription factor activity of mouse brain

**DOI:** 10.1016/j.xpro.2022.101633

**Published:** 2022-08-18

**Authors:** Hajime Yamamoto, Kentaro Abe

**Affiliations:** 1 Lab of Brain Development, Graduate School of Life Sciences, Tohoku University, Katahira 2-1-1, Aobaku, Sendai, Miyagi 980-8577, Japan; 2 Division for the Establishment of Frontier Science, Tohoku University, Katahira 2-1-1, Aobaku, Sendai, Miyagi 980-8577, Japan

**Keywords:** Cell Biology, Developmental biology, Molecular Biology, Gene Expression, Neuroscience

## Abstract

Here, we provide a step-by-step protocol to measure the activities of multiple transcription factors (TFs) in the same mouse brain. This protocol includes a procedure to construct a virus-based TF activity reporter, *in utero* transfection, and PCR-based measurement of TF activity to obtain the transcription factor activity profile (TFAP). Our protocol facilitates a systematic analysis of TF activity of the brain *in vivo* and will aid trans-omics understanding of the molecular mechanism underlying the brain functions.

For complete details on the use and execution of this protocol, please refer to Abe and Abe, (2022).

## Highlights

• Protocol allows quantitative measurement of TF activity in mouse brain
• A protocol for preparation and *in utero* injection of LV-based transcription reporter
• Workflow of behavioral experiment and brain sampling for TF activity measurement

## Abstract (editor highlights)

Publisher’s note: Undertaking any experimental protocol requires adherence to local institutional guidelines for laboratory safety and ethics.

## Graphical abstract

**Figure:**
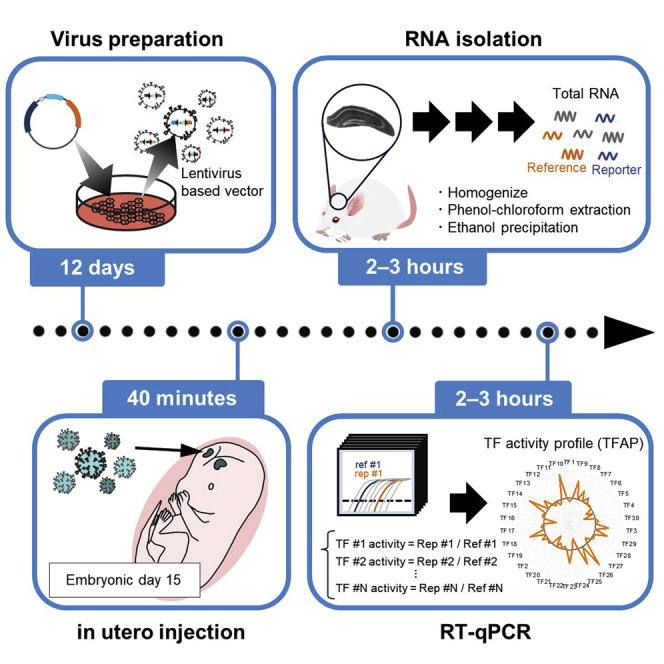

## Before you begin

The identity of cells is established by the joint activity of multiple TFs that are dynamically regulated by various stimuli (Takahashi and Yamanaka, 2006; Vaquerizas et al., 2009; Spitz and Furlong, 2012; Lambert et al., 2018). Thus, quantitative measurement of the activities of various TFs and how those activities change upon experimental stimulation will aid the trans-omics understanding of how cells respond to a variety of biological stimuli. However, it is still challenging to measure the endogenous activity of TF, particularly *in vivo*. Recently, we have reported the measurement of TF activity *in vivo* by using viral-vector-based TF activity reporters (Abe et al., 2015; Abe and Abe, 2022). This protocol describes the detailed procedures used in those studies for measuring the transcription factor activities of multiple TFs in the brain of mice.

### Institutional permissions

The studies using animals need permission from the institutional animal care and use committee (IACUC). The care and experimental manipulation of animals used in this study were reviewed and approved by the IACUC at Tohoku University. The procedures using recombinant DNAs or viral vectors must be conducted in the corresponding biosafety level (BSL) laboratory with proper equipment. For instance, the preparation of lentiviral vectors (LV) and LV-related experiments need a BSL-2 laboratory. Appropriate disinfection is required for discarded materials.

### Preparation of plasmid vector for TF activity reporter viruses

**Timing: 7 days**

**Figure 1:**
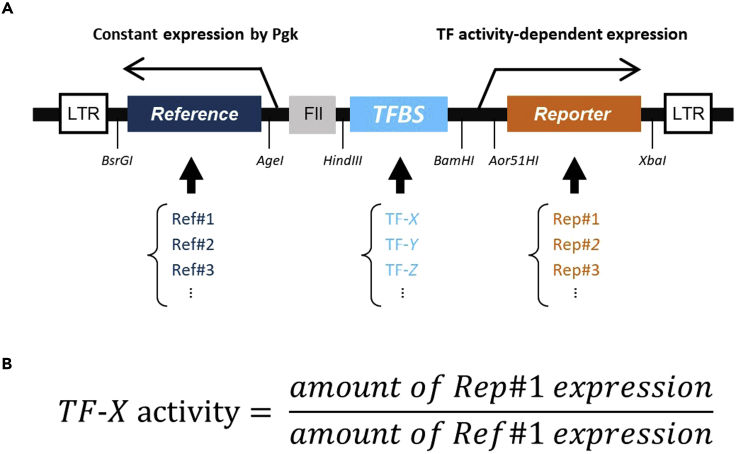
Schematics of the TF activity measurements (A) Schematics of the reporter constructs. For a given TF, its TFBS is associated with a pair of Reference and Reporter genes; for example, the TFBS of a transcription factor “TF-X″ is paired with Ref#1 and Rep#1. FII, insulator; LTR, long terminal repeat. (B) To calculate the activity of a specific TF, the amount of the Rep, quantified by qPCR, was divided by the amount of the corresponding Ref. This procedure results in a robust calculation of endogenous TF activity irrespective of transfection efficiency (Abe et al., 2015).

Our method utilizes LV-based TF activity reporters that can be used for analyzing the activities of endogenous TFs (Abe et al., 2015; Abe and Abe, 2022). Briefly, these viruses express two genes from two independent promoters: a reporter-gene (Rep) from a TF-activity-dependent promoter and a reference-gene (Ref) from a constantly active Pgk (human phosphoglycerate kinase 1) promoter (Figure 1). The TF-activity-dependent promoter consists of transcription factor binding sequences (TFBS) of the corresponding TFs followed by a synthetic minimal-promoter. The TFBS of the TF-activity-dependent promoter can be changed to those of other TFs with verified TFBSs to create a TF activity reporter virus of the TF of interest. The activity of each TF was defined by the ratio of the amount of mRNA of Rep and Ref quantitated by PCR. We usually use six pairs of Rep and Ref genes, but the number of reporter genes can be extended if the PCR primer independently amplifies each Rep gene with no cross-reactivity.

1. Replace the TFBS of the plasmid pLV-TFrep#1–#6 with the TFBS of the TF of interest.
  a. Synthesize TFBS of the target TF, flanked by HindIII and BamHI site.
  b. Subclone the sequence between HindIII and BamHI site of pLV-TFrep#1-#6.

**CRITICAL:** It is necessary to confirm beforehand that the TFBS reflects the activity of TF of your interest onto the expression of the reporter gene. An *in vitro* luciferase reporter assay is suitable for such confirmation (de Wet et al., 1987; Alam and Cook, 1990).

2. Prepare enough amount of the transfection grade plasmids, diluted to 1 μg / μL.

***Note:*** 18 μg of transfection grade plasmid is necessary for transfecting one dish of HEK293T cells on 15 cm dish. The base plasmid#1–#6 can be obtained from Addgene (#182054, #182055, #182056, #182057, #182058, #182059).

**Pause point:** Plasmids can be stored at –80°C for years.

### Preparation of viral vectors

**Timing: 5 days**

3. Transfect one dish of HEK293T cells with plasmid DNAs by Polyethyleneimine (PEI) mediated transfection. The quantity of reagent below is required for transfection of 1 dish.
  a. Mix 18 μg of reporter constructs with 20 μg of the 3rd generation lentivirus packing and envelope plasmids (a mixture of pRSV-Rev, 5.3 μg; pMDLg/RRE, 11 μg; pMD2.G, 3.6 μg).
  b. Dilute the plasmids with 167 μL of Opti-MEM and mix with 300 μL of PEI in 540 μL of Opti-MEM I.
  c. Wait for 20 min and add 1 mL of the mixture to the cells.
  d. After 16 h of transfection, change the medium and maintain the cells with Dulbecco’s Modified Eagle Medium (DMEM) with 10% Fetal bovine serum (FBS) and Penicillin / Streptomycin.
4. Harvest the virus-containing medium 48 h after the transfection. Filtrate the collected medium through a 0.45 μm pore filter.
5. Pellet the viruses in the medium by adding 4 × Virus Condensation Solution (see materials and equipment) at the final concentration of 1 × and store them at 4°C for 16 h.
6. Centrifuge at 4,350 × *g* at 4°C and dissolve the virus pellet in 1 mL of PBS.
7. Overlay the suspended virus solution on the 20% Sucrose/PBS solution.
8. Centrifuge at 15,000 × *g* at 4°C and dissolve the pellet in 50 μL of PBS.
9. Sub-divide the PBS dissolved virus solution into 4 μL and stock them at –80°C.
10. Check the titer of the virus by transfecting them to HEK293T cells and quantifying the genome integration of the transgene by quantitative PCR (qPCR). For our hands, a minimum of 2.0 × 10^10^ infectious unit / mL is required for stable measurements of TF activity in vivo.

**CRITICAL:** Avoid repeated freeze and thaw of the virus solution to keep its titer.

**Pause point:** Viruses can be stored at –80°C for years.

***Note:*** Detailed procedures of production, purification, and titration of LV are described before (Kutner et al., 2009; Brown et al., 2020).

### Preparation for *in utero* injection

**Timing: 60 min**

11. Obtain a pregnant female mouse having E15 embryos.

**CRITICAL:** If a pregnant female mouse is purchased from a commercial breeder, keep it in the animal facility to acclimatize at least 3 days before the surgery. This procedure reduces delivery failure due to stress.

***Note:*** We recommend using the mice of the ICR strain for *in utero* injection because this strain yields more pups than other major strains and the dams are more robust to surgical manipulation. Different strains, such as C57BL/6, can be used without modifications to this protocol.

**Figure 2:**
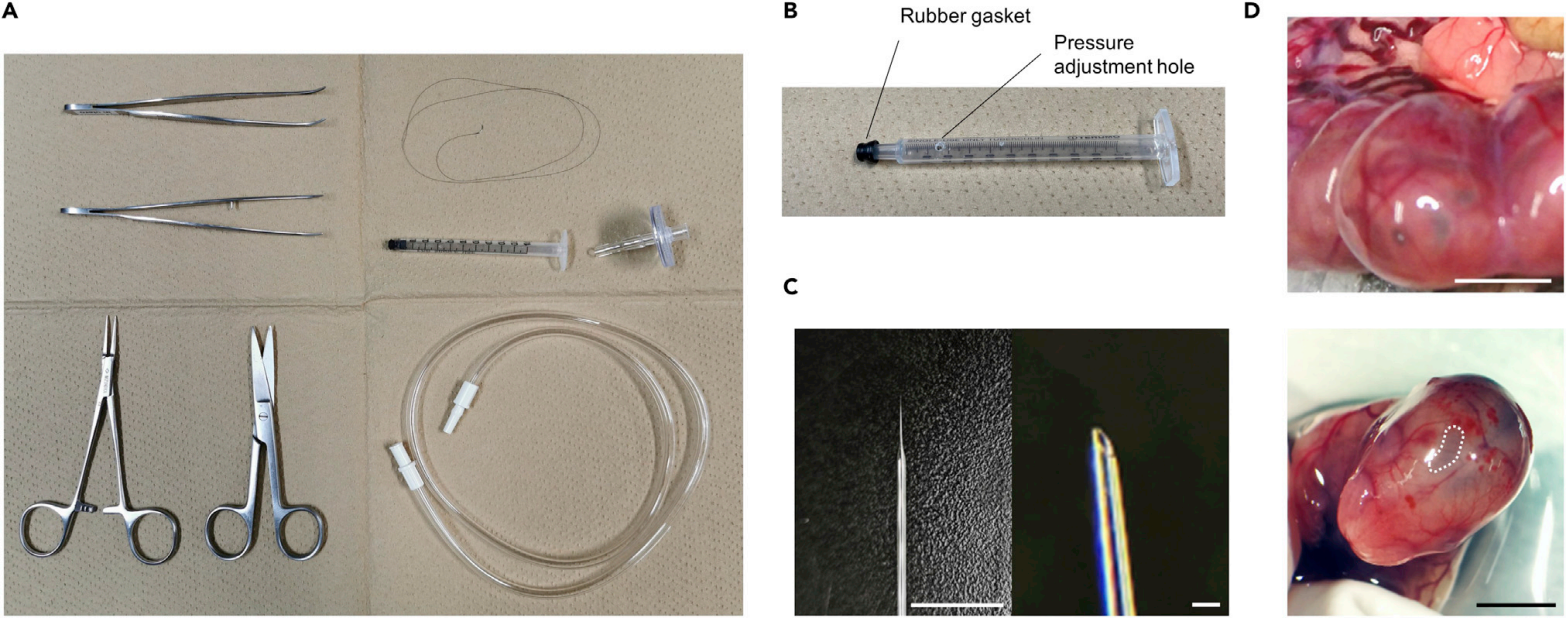
Schematics of the *in utero* LV injection (A) Surgical tools for the surgery. All instruments are sterilized with 70% ethanol before surgery. (B) Needle holder. The injection needle is attached to a needle holder which was made from a syringe on which a rubber gasket was glued. The holder is connected to an extension tube through a membrane filter. Sealing the hole with the experimenter’s finger aid the adjustment of pressure applied to the needle tip. (C) The tip of the glass needle which was polished by a micropipette beveler. Scale bar, 10 mm (left) and 100 μm (right). (D) Top: A virus-injected embryo *in utero*. Bottom: An image of an embryo removed from the uterus. The white dotted area on the right hemisphere indicates the lateral ventricle where the virus solution was injected. Scale bar, 10 mm (top) and 5 mm (bottom).
13. Maintain sterilized ∼10 mL of PBS at 40°C–42°C with a heat-block incubator.
14. Create injection needles by heat-elongation of a fine glass capillary with a micropipette puller at 62°C and polish their tip to 45° with a micropipette beveler (Figure 2C).
15. Load the following virus mixture into the injection needle.
  a. Mix each TF activity reporter virus for TFs of interest and scale up with Cortex Buffer (see materials and equipment). Approximately 1 μL of the virus mixture solution is required for each embryo.
  b. Add 0.1% Fast Green (final concentration 0.002%) to aid visualization of the virus mixture solution during the injection into embryos.
16. Attach the glass needle to a needle holder made from a syringe on which a rubber gasket was glued (Figure 2B). Connect the holder to a pressure source.

***Note:*** We usually mix up to six TF activity reporter viruses bearing six pairs of Ref and Rep. Using viruses with the titer of 2.0 × 10^10^–8.0 × 10^12^ infectious unit / mL is recommended. Since the titer of lentivirus drops upon storage at 4°C or freeze and thaw process, we recommend preparing the required amount of virus from frozen stock solution sub-divided in 4 μL, immediately before the injection.

12. Disinfect the surgical instruments with 70% ethanol (Figures 2A and 2B).

## Key resources table

**Table:** 

REAGENT or RESOURCE	SOURCE	IDENTIFIER
**Antibodies**		

Anti-Flag, M2, 1:2000	Sigma-Aldrich	Cat#F1804, RRID: AB_262044

**Bacterial and virus strains**		

LV-Rep/Ref	(Abe and Abe, 2022)	N/A

**Chemicals, peptides, and recombinant proteins**		

2-Mercaptoethanol	Wako	Cat#133-14571
2-Propanol	Wako	Cat#163-04846
8-Hydroxyquinoline	Nacalai Tesque	Cat#18912-52
Butorphanol tartrate	Meiji Seika Pharma	Vetorphale
Calcium chloride	Wako	Cat#030-25045
Chloroform	Wako	Cat#035-02616
D(+)-Glucose	Wako	Cat#046-31175
4′,6-Diamidino-2-phenylindole (DAPI)	Dojindo Molecular Technologies	Cat#D523
Diethylpyrocarbonate	Nacalai Tesque	Cat#12311-86
Dulbecco’s Modified Eagle Medium (DMEM)	Wako	Cat#044-29765
Ethanol	Nacalai Tesque	Cat#14710-25
Fatal bovine serum (FBS)	Biowest	Cat#S1530
FCF Fast Green	Nacalai Tesque	Cat#15939-54
GoTaq qPCR Master Mix	Promega	Cat# A6002
Guanidinium thiocyanate	Nacalai Tesque	Cat#06287-45
HEPES	Nacalai Tesque	Cat#02443-05
Magnesium sulfate	Wako	Cat#034-00925
Medetomidine hydrochloride	All Japan pharma	Domitol
Midazolam	Astellas Pharma	Dormicum
N-Lauroylsarcosine sodium salt solution	Nacalai Tesque	Cat#20135-14
Opti-MEM I	Thermo Fisher Scientific	Cat# 31985070
PBS	N/A	N/A
PEI-MAX	Polysciences	Cat# 24765
Penicillin / Streptomycin	Wako	Cat#168-23191
Phenol	Nacalai Tesque	Cat#26728-45
Polyethylene glycol 6,000	Nacalai Tesque	Cat#28254-85
Potassium chloride	Wako	Cat#160-03555
ReverTra Ace qPCR RT Master Mix with gDNA remover	Toyobo	Cat#FSQ-301
Sodium acetate	Wako	Cat#199-01085
Sodium chloride	Wako	Cat#198-01675
Sucrose	Wako	Cat#196-00015
Sodium citrate	Wako	Cat#198-01795

**Experimental models: Organisms/strains**		

Mouse: Slc:ICR (Embryonic day 15, 16-day-old, and 8-week-old male mice)	SLC Japan	N/A

**Oligonucleotides**		

RT-primer	(Abe and Abe, 2022)	See Primer List in Table 1.
qPCR-primer	(Abe and Abe, 2022)	See Primer List in Table 2.

**Recombinant DNA**		

pLV-Rep/Ref	(Abe and Abe, 2022) Addgene	#182054, #182055, #182056, #182057, #182058, #182059
pRSV-Rev	Addgene	Cat#12253
pMD2.G	Addgene	Cat#12259
pMDLg/pRRE	Addgene	Cat#12251

**Other**		

0.45 μm pore filter	Nalgene	Cat#121-0045
Artery forceps	Frigz	Cat#E560-845
Beads mill homogenizer	Bio-Medical Science	Cat#45-1291
Dissecting scissors	Frigz	Cat#D340-476
Extension tube	Terumo	Cat#7-4621-06
Fine glass capillaries	Harvard Apparatus	Cat#30-0038
Heating pad	Vivaria	Cat#LP-817
LightCycler 480 Multi-well Plate 384, white	Roche Diagnostics	Cat#04729749001
LightCycler 480 Sealing Foil	Roche Diagnostics	Cat#04729757001
LightCycler 480 System II	Roche Diagnostics	Cat#05015243001
Low adsorption microtubes	Treff	Cat#96.07246.9.01
Membrane filter	ADVANTEC	Cat# 63-1237-28
Micropipette beveler	Narishige	Cat#EG-45
Micropipette puller	Narishige	Cat#PC-100
Screw cap tube, 2.0 mL	Simport	Cat#T330-7
Stainless steel beads, 5 mm diameter	AS ONE	Cat#2-9244-05
Surgical suture	Bear Medic	Cat#61-9190-12
Syringe	Terumo	Cat#1-4908-01
Thermal cycler	Bio-Rad	Cat#1861096
Tissue forceps	Frigz	Cat#A049-0751

.

**Table 1:** RT-PCR Primer list

Target	Sequence
Ref-UTR#1	CCACATAGCGTAAAAGGAGCAAC
Ref-UTR#2	TGGTTGCTGTCTCTTTATGA
Ref-UTR#3	CATTTTCTCCTCCTTGTAT
Rep-C-term#1	GCAAGCAGCAGGGTGTCTATC
Rep-C-term#2	ACAGGGACAGCAGAAA

**Table 2:** qPCR Primer list

Target	Forward sequence (5′–3′)	Reverse sequence (5′–3′)
Ref#1	TACTGCCCAAGAAGACCGAGA	CCACATAGCGTAAAAGGAGCAAC
Rep#1	AACTGCTTCATCCACAACTCCA	ACGGGCTTCTCGGTCTTGT
Ref#2	TACTAAGGCCATCACCAAGTACACC	CCACATAGCGTAAAAGGAGCAAC
Rep#2	CTGAACCACGACAAGGACTACAAC	TGGAGGGAAGCCGTGAGAA
Ref#3	GGCGAACTCGCGAAACA	CCACATAGCGTAAAAGGAGCAAC
Rep#3	GGAGGAGGATCACAGCAACA	TGGAGGGAAGCCGTGAGAA
Ref#4	CAAAGGCAAGGGCTATCTGG	CCACATAGCGTAAAAGGAGCAAC
Rep#4	GGACGCCACCATCTGTTTCT	TGGAGGGAAGCCGTGAGAA
Ref#5	AGTTGGCTAAGCACGCTGTC	CCACATAGCGTAAAAGGAGCAAC
Rep#5	GACAAGCAGAAGAACGGCATC	TGGAGGGAAGCCGTGAGAA
Ref#6	CCACCCACAGTTTGAGAAGG	CCACATAGCGTAAAAGGAGCAAC
Rep#6	GCCCCGTGATGAAGAAGATG	TGGAGGGAAGCCGTGAGAA

## Materials and equipment

**Table:** 4 × Virus Condensation Solution

Reagent	Final concentration	Amount
Polyethylene glycol 6,000	320 mg/mL	160 g
Sodium chloride (5 M)	400 mM	40 mL
HEPES (1 M, pH = 7.4)	40 mM	20 mL
ddH_2_O	N/A	190 mL
Total		250 mL

***Note:*** The Virus Condensation Solution should be stored at 25°C for up to 3 months.

**Table:** Anesthesia

Reagent	Final concentration	Amount
Medetomidine hydrochloride (1 mg/mL)	30 μg/mL	0.75 mL
Midazolam (5 mg/mL)	400 μg/mL	2.0 mL
Butorphanol tartrate (5 mg/mL)	500 μg/mL	2.5 mL
Sodium chloride (5 M)	118 mM	0.59 mL
ddH_2_O	N/A	19.1 mL
Total		25 mL

***Note:*** The anesthetic solution should be stored at 4°C for up to 3 months and used according to the law and regulation.

**Table:** Cortex Buffer

Reagent	Final concentration	Amount
Sodium chloride (1 M)	125 mM	6.25 mL
Potassium chloride (1 M)	5 mM	0.25 mL
Glucose (1 M)	10 mM	0.5 mL
HEPES (1 M, pH = 7.4)	10 mM	0.5 mL
Magnesium sulfate (1 M)	2 mM	0.1 mL
Calcium chloride (1 M)	2 mM	0.1 mL
ddH_2_O	N/A	42.3 mL
Total		50 mL

***Note:*** Cortex Buffer should be stored at 4°C for up to 3 months.

**Table:** Water-saturated phenol

Reagent	Final concentration	Amount
Phenol (solid)	N/A	500 g
8-Hydroxyquinoline	0.1%	0.5 g
ddH_2_O	N/A	> 300 mL
Total		N/A

***Note:*** Heat a bottle of solid Phenol at 65°C to melt, and mix the reagents above. Mix the solution harshly by shaking the bottle by hand and leave at 4°C at least 48 h before use. Confirm the solution is separated into two-phase and use the bottom phenol layer. Water-saturated phenol should be stored at 4°C for up to 3 months.

**Table:** RNA Extraction Solution

Reagent	Final concentration	Amount
Guanidinium thiocyanate (3.5 M)	1.63 M	22.3 mL
Sodium citrate (0.75 M, pH = 7.0)	11.4 mM	0.73 mL
30% N-Lauroylsarcosine sodium salt solution	0.23%	0.37 mL
2-Mercaptoethanol	0.0033%	0.159 mL
Sodium acetate (3 M, pH = 4.0)	6.25 mM	0.1 mL
Water saturated phenol	N/A	23 mL
Total		47 mL

***Note:*** RNA Extraction Solution can be stored at 4°C for 3 months. Bring to 25°C before use.

## Step-by-step method details

### *In utero* injection of TF activity reporter viruses

**Timing: 40 min per female mouse**

**Figure 3:**
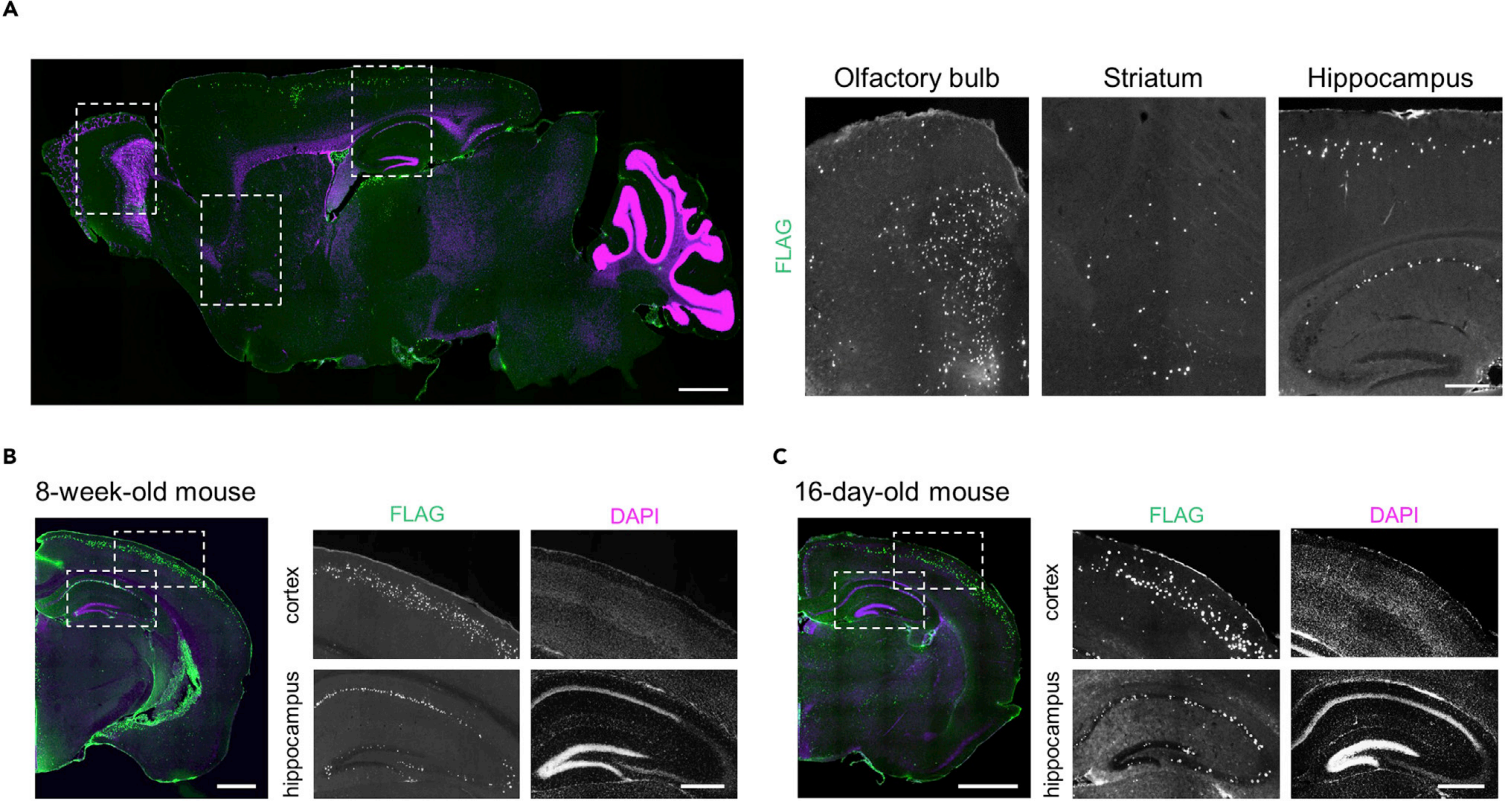
Expected expression of reporter constructs The fluorescent image of the brain sections of mice injected with LV-based reporter at E15, immunostained with an antibody recognizing the Ref gene (anti-Flag-tag, green), shown with 4′,6-diamidino-2-phenylindole (DAPI) staining (magenta). The higher magnification images within the dotted squares are shown on the right. (A) Sagittal brain section of an 8-week-old mouse. Scale bar, 1,000 μm (left) and 500 μm (right). (B) Coronal brain section of an 8-week-old mouse. Scale bar, 1,000 μm (left) and 500 μm (right). (C) Coronal brain section of a 16-day-old mouse. Scale bar, 1,000 μm (left) and 500 μm (right).

For generating the mice which express the TF activity reporter constructs in a defined cell population of their brains, we injected the LV-based TF activity reporters into the lateral ventricle of the intrauterine embryos at E15 (known as *in utero* injection). This method results in the expression of TF activity reporter constructs in the neurons of a wide area of telencephalon such as the cortex, basal ganglia, and hippocampus (Figure 3). The detailed procedures of *in utero* injection of viral vectors at a similar embryonic stage have been described before (Dong et al., 2022). Briefly,

1. Anesthetize a pregnant mouse with an intraperitoneal injection of 500 μL anesthetic (see materials and equipment).
2. Confirm the surgical level of anesthesia. Check the absence of reflection to pinching the paw with forceps.
3. Disinfect the abdomen of the mouse with 70% ethanol.
4. Cut the skin along the midline (2–3 cm) with sterilized scissors.
5. Gently take the embryos out from the abdomen, and hydrate them and the abdominal cavity with warm PBS.
6. Use a flashlight to visualize the embryos and the ventricle of interest, where the injection needs to be administered. If the embryo is not in the optimal position, carefully relocate the head of the embryo with your fingers.
7. Inject the virus solution loaded in the injection needle into the lateral ventricle of each embryo. Approximately 1 μL of the viral mixture is pressure-injected into each embryo by the glass injection needle.
8. Put the embryos back into the abdomen of the female. Fill the abdominal cavity with warm PBS.
9. Suture the abdominal and skin incision.
10. Heat the body with a heating pad until recovery from anesthesia and return to the home cage.

***Note:*** Inject a similar amount of virus into every embryo. Since it is hard to non-invasively quantify the amount of viral infection after birth, failure or variation of viral infection among the siblings will lower the quality of TF-activity measurement and their comparison between the condition.

***Note:*** Analgesic reagents such as medetomidine or butorphanol are included in the anesthesia used in this protocol.

**CRITICAL:** The anesthetic time depends on the anesthesia used. The duration of anesthetic time of the medetomidine-midazolam-butorphanol mixture used in this protocol is reported to be around 40–50 min (Kawai et al., 2011). Practice well to conduct the entire procedure within this time period. Other anesthesia, such as isoflurane, can be used instead. To prevent damage to the embryos and the dam, constantly apply warm PBS to the embryos to avoid dehydration.

### Preforming behavioral stimulation on mice

**Timing: 2–3 h**

This step describes the timing and tips of sacrificing animals for sample preparation. Because our procedure relies on measuring and comparing the TF-activity among different animals across the different conditions, it is important to control the basal activity of mice and their brains. Irregular or aggressive behavior immediately before the sample preparation may cause undesired impacts on the TF-activity measured from such animals. For example, keeping mice individually housed before and during an experiment and performing an experiment at the same time of day for all the subjects will aid the quality of data obtained.

The experiment procedure of sample preparation changes according to what kind of TF activity the experimenter tries to assess. In one case, if the experimenter intended to measure the basal activity of TFs, it is required to keep the animals in a calm situation in their home cages before sacrificing them. In another case, if the experiment is intended to assess the change of TF activity upon experimental stimuli or induced behaviors, it is required to perform the behavioral experiment at exactly the same timing for every animal and wait for the time necessary to reflect the change of TF activity on the expression of Rep. Since each TF has its own time course of activation, which may depend on activation timing and the speed of signal transduction within the cell and so on, it is required to determine and be strict to the timing of behavioral experiment and sample preparation.

11. After the pups are born, execute experiments needed at the desired age.
  a. Before the experiments, keep the mice individually housed for 2–3 days.
  b. Subject behavioral experiment to each mouse.
  c. Wait for the time required to reflect on the TF-activity change.
  d. Sacrifice the mice and promptly proceed to the subsequent RNA purification process.

**CRITICAL:** This process should be performed after confirming that mice are expressing TF activity reporter constructs in the desired cell population. To assess this, we recommend performing immunostaining of brain sections obtained from some siblings. The transfected population can be efficiently evaluated by using the antibodies against the immunostaining tags included in the Ref genes (i.e., Flag, HA, V5, Myc, C-tag, Strep-tag).

***Note:*** The time required to reflect the TF-activity differs across TFs. To measure the basal activity change of TF-activity according to chronic manipulation, such as gene knockout and chronic administration of reagents, no particular experimental stimulation is required. Nevertheless, keeping the mice in the same condition for the same duration before the sample preparation will aid the reproducibility of data.

### Purifying RNA from a tissue sample

**Timing: 2–3 h**

This step describes the procedure for purifying RNA to measure the TF activity. Obtaining total RNA of mice expressing TF activity reporter requires homogenization and purification process; this protocol uses a guanidinium thiocyanate-phenol-chloroform extraction (Chomczynski and Sacchi, 1987). In this protocol, we use a homemade RNA extraction solution, but a commercially available reagent (e.g., TRIZOL, TRI reagent) can be used instead.

12. Prepare RNA Extraction Solution for homogenizing tissue samples. The contents of this solution are listed in the key resources table and materials and equipment. The addition of chloroform at step 20 is required for phenol-chloroform extraction of RNA.
13. Keep a screw cap tube with 800 μL PBS on ice.
14. Euthanize the mouse according to institutional guidelines.
15. Dissect the brain into ice-cold PBS.
16. Dissect the desired brain region under a microscope and collect the tissue (50–150 mg) into the ice-cold PBS in the screw tube.
17. Replace PBS with 1 mL of room temperature RNA extraction solution and put a 5 mm stainless bead within the tube for homogenizing the tissue.
18. Shake the tube with a beads mill homogenizer for 30 s at 3,800 rpm.

**CRITICAL:** To prevent artificial TF-activity change or RNA degradation, always keep the brain and dissected tissue on ice. Promptly proceed with the procedure from the euthanizing of the mouse until the homogenization in RNA extraction solution. We recommend performing this procedure individually for each euthanized animal, paying attention that all the animals are processed at the same time course.

**Pause point:** Homogenized tissue can be stored at –80°C for years before the RNA purification.

19. Keep the samples at room temperature.
20. Add 0.3 mL of chloroform to the homogenized tissue and stir vigorously with a beads mill homogenizer or a vortex mixer for more than 30 s.
21. Store the samples for 3 min at room temperature.
22. Centrifuge at 12,000 × *g* for 15 min at 20°C.
23. Collect ∼500 μL of the supernatant into a new tube.

***Note:*** The opaque middle layer between chloroform and the transparent upper layer contains DNA. Keep the middle layer untouched during the collection of the supernatant from the upper layer. To increase the purity of RNA, the process of chloroform-liquid separation should be repeated one more time. The amount of supernatant collected in the second separation should be less than ∼300 μL.

24. Add an equal volume of 2-propanol and precipitate the total RNA by centrifuging at 12,000 × *g* for 15 min at 4°C.
25. Wash the precipitated RNA with 75% ethanol and centrifuge at 12,000 × *g* for 5 min at 4°C.
26. Dry the RNA pellet and dissolve it with RNase-free water at 55°C.
27. Store the RNA at –80°C.

**CRITICAL:** The recommended RNA concentration is less than 1 μg / μL. Too much total RNA lowers the efficiency of RT-PCR.

***Note:*** All the reagents must be RNase-free. For making solutions, use RNase-free water, such as diethylpyrocarbonate treated water.

**Pause point:** RNA solution can be stored at –80°C for years.

### RT-qPCR for purified RNA

**Timing: 2–3 h**

This step describes the procedure for efficiently synthesizing cDNA of the TF activity reporter gene from the isolated RNA. RT-PCR was performed with ReverTra Ace with gDNA remover kit. To efficiently reverse transcribe the virus-vector-derived reporter genes (Reps and Refs), we use gene-specific primers targeting the sequence common to all Ref and Rep (Table 1) in addition to RT-primers included in the kit.

28. Remove contaminating genome DNAs in the RNA samples by gDNA Eraser included in the kit. Detailed volume and reaction time are described below.

**Table:** Genome removal reaction master mix

Reagents	Final concentration	Volume
Total RNA	N/A	3.3 μL
4 × gDNA Eraser	1 ×	1.33 μL
RNase free water	N/A	0.67 μL

**Table:** Genome removal reaction conditions

Steps	Temperature	Time
Genome DNA removal	37°C	5 min
	4°C	Forever

29. Perform RT-PCR on the RNA samples. Detailed volume and reaction time are described below.

**Table:** RT-PCR reaction master mix

Reagents	Final concentration	Volume
Purified RNA	N/A	5.3 μL
5 × RT Master Mix II	1 ×	1.47 μL
Gene specific RT-primers (mixture of 5 primers)	0.15 μM	0.53 μL

**Table:** RT-PCR conditions

Steps	Temperature	Time
Reverse transcription reaction	37°C	15 min
Reverse transcription reaction	42°C	5 min
Reverse transcription reaction	50°C	5 min
Enzyme inactivation reaction	95°C	5 min
Hold	4°C	Forever

***Note:*** The resultant cDNA can be diluted to 5 × with RNase-free water and used for the subsequent qPCR analysis.

**Pause point:** The cDNAs can be stored at –80°C for months. Avoid repeated freeze and thaw.

30. Quantify the expression of Rep and Ref by qPCR.
  a. Create calibration curves using stepwise dilutions of plasmid DNA of known concentration containing the sequence of the target genes; Reps and Refs.
  b. Prepare qPCR reaction master mix without cDNA according to the following table and dispense 8 μL of the master mix into a PCR plate.
  c. Add 2 μL of cDNA to each well.
  d. Mix the solution well.
  e. Perform qPCR.

***Note:*** This protocol uses the intercalation method with absolute quantification. Detailed volume and reaction time are listed below.

**Table:** qPCR reaction master mix

Reagent	Final concentration	Volume
2 × GoTaq master mix	1×	5 μL
H_2_O	N/A	2.95 μL
Primer set	0.25 μM	0.025 × 2 μL
cDNA	N/A	2 μL

**Table:** PCR cycling conditions

Steps	Temperature	Time	Cycles
Initial Denaturation	95°C	2 min	
Denaturation	95°C	10 s	60 cycles
Annealing and Extension	64°C	55 s	
Melt curve analysis	64°C–95°C		
Hold	12°C	Forever

**CRITICAL:** qPCR to quantify each of the paired genes of Ref and Rep must be conducted simultaneously on a single PCR plate. If multiple TF activity reporters were expressed in the same sample, it is necessary to confirm that the primer-set amplifies the target sequence without nonspecific amplification. For such confirmation, perform PCR on negative control samples that include all the plasmids conveying all the Reps and Refs but the target ones.

31. For each Ref and Rep, calculate the TF activity by normalizing the Rep expression quantity by that of the corresponding Ref for each sample (Figure 1B). Summarize the activity data to obtain the TFAP, which aids a comprehensive understanding of the activities of multiple TFs in the mouse brain.

**CRITICAL:** Absolute quantification is necessary to determine the exact amount of cDNA of the target molecule. We typically obtain 1,000–10,000 molecules of Ref genes per 2 μL of cDNA used for qPCR. A low amount of Ref gene expression will distort the calculation of TF activities.

## Expected outcomes

Measuring the TF activity in a defined cell population is crucial to obtaining TFAP because the TF activities may largely differ depending on the cell types (Abe and Abe, 2022). By *in utero* injection of LV at E15, the predominant population of the cells that express the transgene are the olfactory bulb, cortex, hippocampus, and striatum (Figure 3A). The expression can be stably observed from at least 16-day-old throughout the adult stages without significant changes in the expression level and cell population (Figures 3B and 3C). The granule cells of the olfactory bulb, excitatory neurons in layer II/III of the cortex, the CA1 pyramidal neurons in the hippocampus, and the medium spiny neurons in the striatum are the predominant populations that express the transgenes. Thus, by dissecting those tissue and collecting RNAs from them, it is possible to obtain TFAP of the defined cell population. The population expressing the reporter can be changed by injecting viruses at different embryonic stages.

**Figure 4:**
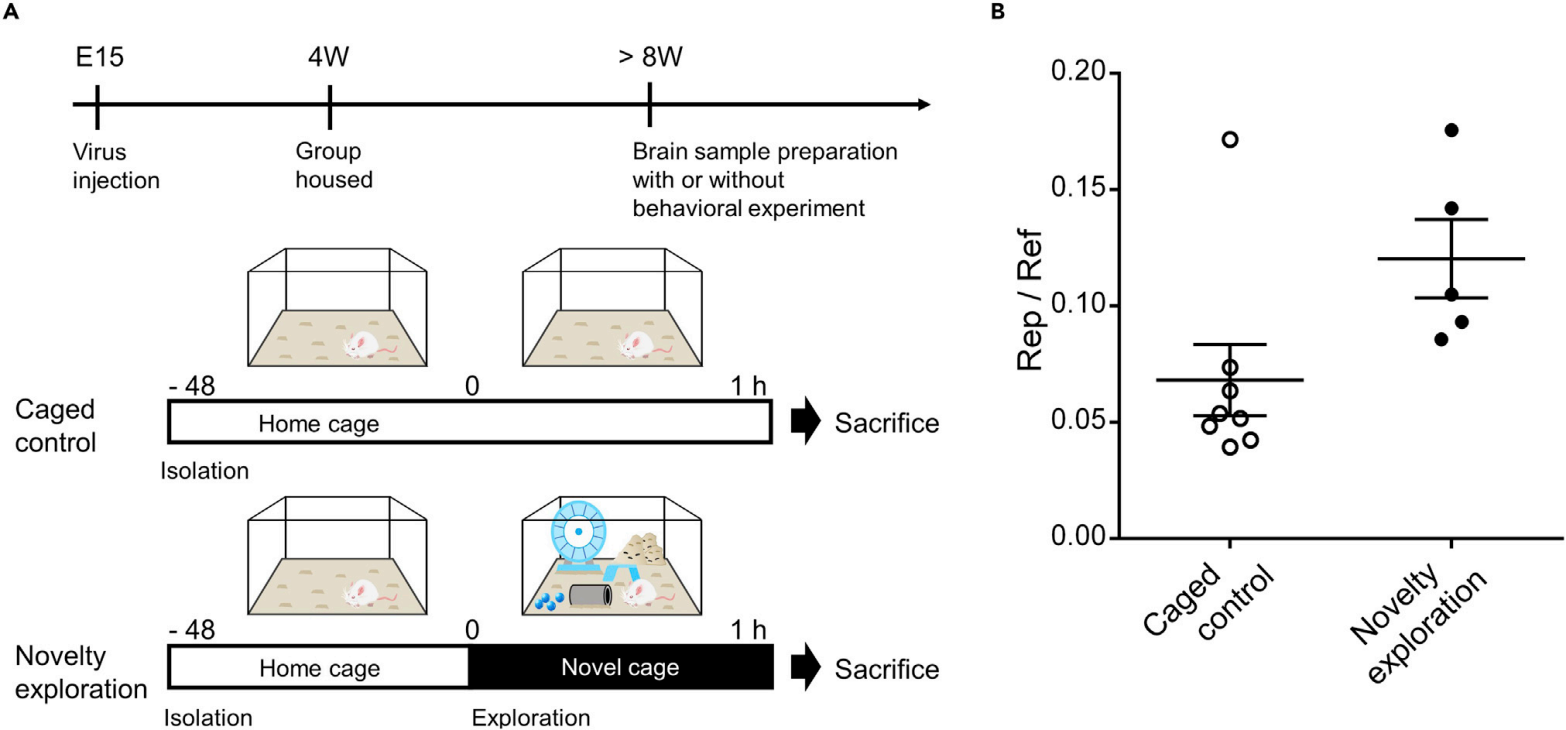
Example of the behavior-induced TF activity change (A) Schematical diagram of the example time-course of measuring the TF activity change induced by behavioral stimulation. Mice expressing TF activity reporter constructs were isolated for 48 h and experienced a novelty exploration. Mice were sacrificed 1 h after the start of exploration. (B) The activity of Arc promoter in the brain of mice experienced different environments. Graph shows mean ± SEM (n = 8, 5).

The TFs in the brain show dynamic changes in their activity. The method described in this protocol allows a quantitative analysis of the change in TF activity induced by behavioral experiments (Abe and Abe, 2022). For example, activity-induced immediate early gene expression was induced within 30 min of novelty exploration in mice (Ramanan et al., 2005). We measured the activity of the Arc promoter during the 60 min of the novelty exploration (Figure 4). Such analysis of the change of TF activity induced by behavior may aid our understanding of how the brain changes according to the environment.

## Limitations

The success of robust measurement of TF-activity is mainly dependent on the efficiency and the reproducibility of the expression of the reporter constructs. The accuracy of *in utero* injection and the developmental stage of manipulation will affect the cell population that expresses the reporter constructs. By using *in utero* injection at E15, cells in the deeper layer of the cortex or region where neurogenesis occurs outside the lateral ventricle at this stage, such as midbrain and hindbrain regions, are less likely to be infected. For studies assessing the TF-activity change in the areas not optimal for expression, it is necessary to individually inject directly into the desired brain region of the adult mice.

## Troubleshooting

### Problem 1

High abortion rate of pregnant females after the surgery. Related to step 11 in “before you begin”.

### Potential solution

Stress and surgical damage have a negative impact on pregnancy and litter viability. Acclimating mice to your laboratory environment at least 3 days prior to *in utero* injection. Performing the surgery quickly (within 40 min) is also essential.

### Problem 2

Abnormal development of virus-injected pups. Related to step 7 in “step-by-step method details”.

### Potential solution

The amount of virus injected should be reduced. We usually mix and inject up to six reporter viruses in 1 μL viral mixture per embryo. Using viruses with the titer of 2.0 × 10^10^–8.0 × 10^12^ infectious unit / mL is recommended. Injecting too much solution per embryo or too much viral particle as a whole often causes abnormal development of the pups.

### Problem 3

Lack of reproducibility of the cell population expressing TF reporters. Related to step 7 in “step-by-step method details”.

### Potential solution

The instability of *in utero* injection techniques results in the lack of reproducibility of the infected cell population. This results in non-reproducible results or no change in TFAP. Practice *in utero* injection procedure to be skilled enough to obtain stable expression of TF reporter constructs in every mouse.

### Problem 4

Low expression of reporter genes is detected by qPCR. Related to step 30 in “step-by-step method details”.

### Potential solution

Robust calculation of TF-activity needs enough amount of Rep and Ref expression. Therefore, strictly adhere to reagent volume, temperature, and reaction time. If this does not resolve the problem, increasing the primer concentration in qPCR is recommended.

### Problem 5

No change in TF activity is observed among the experimental groups. Related to step 31 in “step-by-step method details”.

### Potential solution

There are time lags between the exposure to the stimulus and its resultant change in TF activity. The time course of TF activity change is not necessarily the same as the time course of related gene expression. This is because TF activities are often feedback-regulated by their down-stream genes or by cell-signaling pathways. Experimenters should investigate the timing of tissue collection for their own purpose.

## Resource availability

### Lead contact

Further information and requests for resources and reagents should be directed to and will be fulfilled by the lead contact, Kentaro Abe (k.abe@tohoku.ac.jp).

### Materials availability

This study did not generate new unique reagents, cell, or mouse lines.

## Data Availability

This study did not generate a new dataset.
